
JOURNAL INFORMATION
==============================
NLM Title Abbreviation: ScientificWorldJournal
Journal ID: ScientificWorldJournal
NLM Journal ID: 101131163
Journal ID: TSWJ

The Scientific World Journal

ISSN: 2356-6140
EISSN: 1537-744X
Publisher: Wiley

ARTICLE INFORMATION
==============================
PMCID: PMC6009400
PMID: 29973840
DOI: 10.1100/tsw.2002.118
Article ID: 794042
Article version: 1
Subjects: Editorial

Selected Peer-Reviewed Papers from the Arsenic Session of the 6th International Conference on the Biogeochemistry of Trace Elements, July 29–August 2, 2001, Guelph, Ontario, Canada

Allen Anne Vindenes *
TheScientificWorld, UK
*Anne Vindenes Allen: aallen@thescientificworld.com
Publication date: 2002
Electronic publication date: 2002 Jan 16
Volume: 2
First page: 134
Last page: 135
Received 2002 Jan 8; Accepted 2002 Jan 10
Copyright: Copyright © 2002 Anne Vindenes Allen.
Copyright year: 2002
License: This is an open access article distributed under the Creative Commons Attribution License, which permits unrestricted use, distribution, and reproduction in any medium, provided the original work is properly cited.
License URL: https://creativecommons.org/licenses/by/3.0/

Keywords: ecotoxicology, bioavailability, adsorption reactions on soils and sediments, chemical speciation and bioavailability, arsenic fate and transport, human and animal health

==============================
Publisher's Note
Arsenic: A Themed Collection of Papers from the 6th International Conference on the 21st International Carbohydrate Symposium
*TheScientificWorldJOURNAL* (2002) 2, 134–135
ISSN 1537-744X; DOI 10.1100/tsw.2002.118

The Scientific World
www.thescientificworld.com

### Selected Peer-Reviewed Papers from the Arsenic Session of the 6th International Conference on the Biogeochemistry of Trace Elements, July 29 – August 2, 2001, Guelph, Ontario, Canada

Anne Vindenes Allen
*TheScientificWorld U.K.*

*Received January 8, 2002; Accepted January 10, 2002; Published January 16, 2002*

**KEY WORDS:** ecotoxicology, bioavailability, adsorption reactions on soils and sediments, chemical speciation and bioavailability, arsenic fate and transport, human and animal health

**DOMAINS:** environmental chemistry, terrestrial environmental toxicology, soil systems, toxicology, bioremediation and bioavailability

The International Conference on the Biogeochemistry of Trace Elements (ICOBTE) was held at the University of Guelph from July 29 to August 2, 2001. This highly successful conference series is held every 2 years, and in 2001 over 600 delegates from over 50 countries from around the world listened to over 300 oral presentations and viewed over 300 poster presentations during the 4-day event. Lead sponsor was Ontario Power Generation; other supporters included Soil Science Society of America, Environment Canada, University of Guelph, The Royal Bank of Canada, INCO Ltd., Rio Algom Ltd., Falconbridge Ltd. ,International Lead and Zinc Research Organization (ILZRO), Inc., Varian Canada Inc., Canadian Society of Soil Science, Mandel Scientific Co. Ltd., and the International Society of Trace Element Biogeochemistry.

Conference themes dealt with many trace element issues including: ecotoxicology; bioavailability; adsorption reactions on soils and sediments; chemical speciation and bioavailability; arsenic and mercury fate and transport; and human and animal health. In addition, ten Special Symposia sessions were organized by world-renowned trace element specialists and included topics such as: biosorption; natural attenuation in soils; metal speciation and bioavailability; phytoremediation; geochemical surface controls on trace element fate; and temporal trends of trace metals in biota. Each day the conference hosted poster sessions that complimented the oral presentations.

TheScientificWorld is pleased to announce the forthcoming publication of peer-reviewed research papers from the session on Arsenic in *TheScientificWorldJOURNAL* under the Guest Editorship of Dr. Muhammad Sadiq. Dr. Sadiq of the Department of Land Resource Science, University of Guelph was Conference Co-Chair along with Dr. Les Evans (Chair) and Dr. Beverly Hale (Co-Chair), also of the University of Guelph. Papers will be published immediately upon peer-review and acceptance to ensure rapid international dissemination of the research

Email: aallen@thescientificworld.com
©2002 with author.

represented. The Publisher wishes to acknowledge the contribution of Dr. Sadiq, as well as the efforts of the authors and reviewers, in bringing together this collection.

**Anne Vindenes Allen**
Senior Editorial Director – Europe
TheScientificWorld

**This article should be referenced as follows:**

Allen, A.V. (2002) Selected peer-reviewed papers from the Arsenic session of the $6^{th}$ International Conference on the Biogeochemistry of Trace Elements, July 29 – August 2, 2001, Guelph, Ontario, Canada. In Arsenic: A Themed Collection of Papers from the $6^{th}$ International Conference on the $21^{st}$ International Carbohydrate Symposium. *TheScientificWorldJOURNAL* **2**, 134–135.
